# Pulmonary Transit Time Can Be Accurately Quantified From Non‐Arterial Input Function Series in First‐Pass Cardiac Perfusion MRI

**DOI:** 10.1002/mrm.70190

**Published:** 2025-11-23

**Authors:** Mingyue Zhao, Lexiaozi Fan, Kyungpyo Hong, Benjamin H. Freed, Jacqueline Urban, Kelvin Chow, Li‐Yueh Hsu, Daniel C. Lee, Daniel Kim

**Affiliations:** ^1^ Department of Radiology Northwestern University Feinberg School of Medicine Chicago Illinois USA; ^2^ Department of Biomedical Engineering Northwestern University Evanston Illinois USA; ^3^ Division of Cardiology, Department of Medicine Northwestern University Feinberg School of Medicine Chicago Illinois USA; ^4^ Cardiovascular MR R&D, Siemens Medical Solutions USA Chicago Illinois USA; ^5^ National Heart Lung and Blood Institute, National Institutes of Health Bethesda Maryland USA

**Keywords:** area under the curve, curve fitting, non‐arterial input function images, peak to peak, pulmonary transit time

## Abstract

**Purpose:**

Previous studies suggested that arterial‐input‐function (AIF) images are necessary to avoid signal saturation for pulmonary transit time (PTT) measurements. This study challenges that notion by investigating whether PTT can be accurately measured using blood pool signals from myocardial enhancement (non‐AIF) images during resting first‐pass perfusion MRI.

**Methods:**

This retrospective study included 108 patients, 47 scanned using a research radial perfusion sequence, and 61 scanned with a prototype Cartesian quantitative perfusion (qPerf) sequence with inline PTT calculation. An automated pipeline was developed to compute PTT measurements from blood pool signals extracted from both AIF and non‐AIF images, as well as from gadolinium concentration curves of the AIF images, using three methods: peak‐to‐peak timing, area‐under‐the‐curve (AUC) derived centroids, and curve‐fitting derived centroids.

**Results:**

Peak‐to‐peak PTT showed low bias (0.10–0.56 s; [1.4%–7.4%] of the mean), wide limits of agreement (5.71–7.42 s; [74.5%–109.8%]). In contrast, centroid‐based methods, using both AUC and curve‐fitting approaches, consistently yielded low bias (< 0.49 s; < 6.3%), narrower limits of agreement (1.83–2.84 s; 25.2%–36.8%). These findings indicate that centroid methods offer more precise and reliable PTT estimation across both radial perfusion and qPerf datasets.

**Conclusions:**

PTT can be derived accurately from non‐AIF images using either the AUC or curve‐fitting centroid‐to‐centroid method.

## 1 Introduction

Pulmonary transit time (PTT), defined as the time it takes for a bolus of blood to travel from the right ventricle (RV) through the pulmonary circulation to the left ventricle (LV), is a measure of cardiopulmonary function, pulmonary blood volume, and pulmonary vascular resistance. It has been shown to predict major cardiovascular events in a variety of heart diseases [1, 2, 3, 4]. Multiple techniques have been proposed for calculating PTT; however, to our knowledge, there is currently no standardized reference. PTT could be estimated by invasive techniques such as the indicator dilution method via cardiac catheterization [5], as well as non‐invasive modalities such as radionuclide imaging [6], computed tomography [7], contrast‐enhanced echocardiography [8]. More recently, first‐pass cardiac perfusion MRI has been proposed as a non‐invasive method for estimating PTT by analyzing the gadolinium concentration ([Gd]) time curves in arterial input function (AIF) [9, 10].

In contrast‐enhanced cardiac MRI protocols, resting first‐pass perfusion imaging can be readily incorporated without substantially disrupting clinical workflow, enabling routine PTT quantification. The simplest approach to measure PTT from first‐pass cardiac perfusion MRI is to calculate the time interval between the RV and LV cavity peaks. One major limitation of this method is its susceptibility to noise and image artifacts, which may distort the identification of the peak locations. A more reliable alternative is to calculate the time interval between the centroids of the RV and LV cavity curves. Seraphim et al. [3] recently developed and validated a curve fitting method to calculate the centroid of fitted curves, while Nelsson et al. [11] proposed deriving the centroid from the area under the curve (AUC) of the first‐passage curve. For these approaches, it is generally preferable to measure PTT from gadolinium concentration ([Gd])‐time curves from arterial input function (AIF) images that are relatively insensitive to signal saturation and distortion [4]. However, many hospitals may lack access to a dual‐imaging pulse sequence or may not have AIF images available as part of a dual‐imaging protocol [3, 12]. Moreover, even when AIF images are available, signal‐to‐[Gd] conversion can be hindered by the lack of knowledge regarding specific sequence parameters, such as the exact repetition time between radio‐frequency excitation pulses and saturation recovery time (TS), which may not be readily accessible in the Digital Imaging and Communications in Medicine (DICOM) headers.

This study challenges the notion that AIF images are necessary to accurately quantify PTT. For this reason, this study investigated whether PTT can be accurately measured using blood pool signals from myocardial enhancement (non‐AIF) images during resting first‐pass perfusion MRI, even without converting signal to [Gd]. We developed an automated pipeline for PTT calculation using both AIF and non‐AIF images for three commonly used techniques: peak‐to‐peak timing, AUC‐derived centroids, and curve‐fitting‐based centroids. The feasibility and reliability of PTT measurements using signal intensity curves from non‐AIF images are systematically evaluated using our research radial perfusion data and further validated against a prototype pulse sequence with inline calculation of PTT derived from the AIF‐based [Gd] time curves using the centroid‐to‐centroid method.

## 2 Methods

### 2.1 Study Population

The study population included two different retrospective cohorts. The first retrospective cohort included 28 consecutive COVID patients (mean age = 48 ± 17 years, 15 males; 18 patients with acute COVID, 10 patients with long COVID) enrolled in a study phenotyping cardiovascular abnormalities due to COVID and 19 consecutive patients (mean age = 57 ± 15 years, 11 males) with chronic thromboembolic pulmonary hypertension (CTEPH) enrolled in a study characterizing right ventricular ischemia. Both studies used a radial pulse sequence, which enables simultaneous reconstruction of AIF and non‐AIF images from the same radial k‐space data [13]. The second retrospective cohort included 61 consecutive patients (age = 57 ± 17 years, 34 males) with suspected coronary artery disease who underwent a clinical stress‐rest perfusion scan using a prototype steady‐state free precession quantitative perfusion (qPerf) dual‐imaging sequence [14]. Exclusion criteria include contraindications for MRI imaging (e.g., metal clips, intracranial clips), pregnancy, severe claustrophobia, and chronic kidney disease with estimated glomerular filtration rate (eGFR) < 45. The relevant patient demographics for both cohorts are summarized in Table S1. All patients in the first cohort provided informed written consent for the parent study and agreed to future use of their data. This study was performed in accordance with protocols approved by our institutional review board and was Health Insurance Portability and Accountability Act (HIPAA) compliant.

### 2.2 MRI Hardware

MRI scans were conducted on three 1.5 T whole‐body MRI scanners (MAGNETOM Aera, Avanto, and Sola, Siemens Healthineers, Erlangen, Germany), equipped with a gradient system capable of achieving a maximum gradient strength of 45 mT/m and a maximum slew rate of 200 T/m/s. A body coil was used for radio‐frequency excitation. Both body matrix and spine coil arrays were used for signal reception.

### 2.3 Pulse Sequence

The relevant pulse sequence parameters for the qPerf and radial pulse sequences are summarized in Table S2. Each perfusion scan was performed with the administration of 0.075 mmol/kg of gadobutrol (Gadavist, Bayer HealthCare Whippany, USA) at 3 mL/s via a power injector.

### 2.4 Image Reconstruction

The radial perfusion images were reconstructed off‐line using Golden‐angle radial MRI (GRASP) (i.e., compressed sensing) [15] on a GPU workstation (Tesla V100 32GB memory, NVIDIA, Santa Clara, California, USA; Xeon E5‐2620 v4 128 GB memory, Intel Corporation, Santa Clara, California, USA) equipped with MATLAB (R2024b, The MathWorks Inc., Natick, MA, USA) running on a Linux operating system (Ubuntu16.04). To accelerate the image reconstruction, coil compression was used to generate eight virtual coils [16], and gpuNUFFT was applied throughout the reconstruction pipeline [17]. As described previously, we used the K‐space Weighted Image Contrast (KWIC) [18] filter to maintain only the center of the first radial spoke to achieve effective TS of 10 ms for the AIF image reconstruction. For the non‐AIF image reconstruction, we excluded the first 12 radial spokes and maintained the center of the last five radial spokes only (i.e., effective TS = 119 ms) to maximize the signal‐to‐noise ratio [19]. Temporal total variation (TTV) and temporal principal component analysis (TPCA) were used as two orthogonal sparsifying transforms and non‐linear conjugate gradient with back‐tracking line search were used as the optimization algorithm with 30 iterations. The normalized regularization weights for TTV and TPCA were set at 0.3 and 0.4 of the median signals of the time‐average image, respectively. For more details on GRASP reconstruction, please refer to [19].

The prototype qPerf images were reconstructed and analyzed inline using the Gadgetron [20] implemented in Siemens Framework for Image Reconstruction Environments (FIRE) [21, 22]. The inline analysis pipeline included PTT measurement derived from AIF images using the centroid‐to‐centroid lognormal curve fitting method on [Gd]‐time curves [23]. However, although the qPerf sequence employs a dual‐imaging protocol with AIF acquisition, the AIF images were not saved by the vendor reconstruction pipeline. Therefore, when using the prototype as the reference standard to validate our approach, only non‐AIF images were accessible and used.

### 2.5 PTT Measurements

**FIGURE 1:**
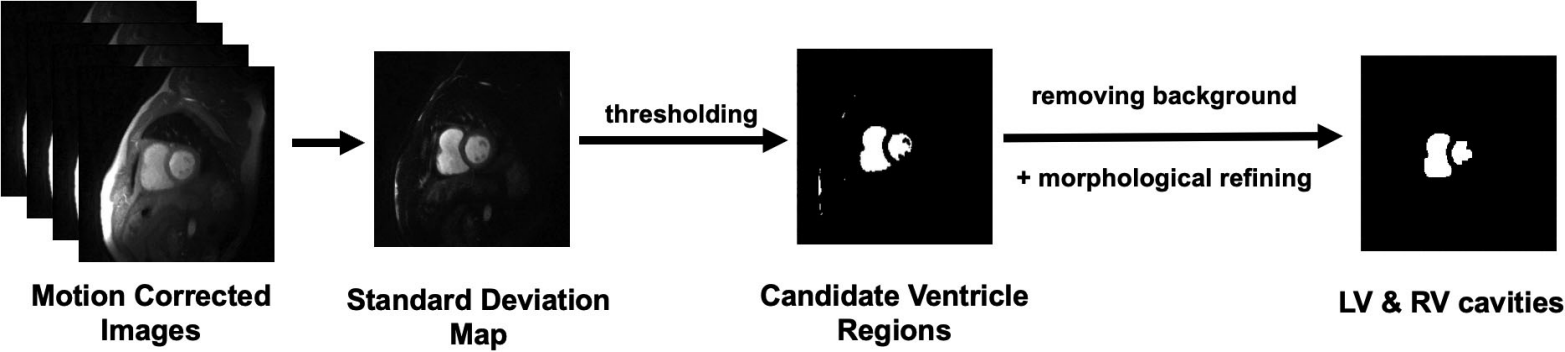
A schematic illustrating the workflow to automatically segment the RV and LV cavities. Following motion correction, candidate ventricular regions are detected by thresholding a standard deviation map. These regions are then refined to isolate the ventricular cavities. LV, Left ventricular; RV, Right ventricular.

Only resting perfusion images were used to calculate PTT. Both the AIF and non‐AIF radial perfusion images were first motion corrected using a previously described method [24]. The prototype qPerf images were motion corrected using the corresponding inline approach embedded in the qPerf WIP. RV and LV signals were then extracted using a modified automated cavity detection algorithm [25], as illustrated in Figure 1. The procedure included the following steps: (a) calculating a pixel‐wise temporal standard deviation (SD) map from the motion corrected dynamic images (excluding proton density frames); (b) selecting candidate ventricle regions by thresholding the SD map at mean + 2 SDs for radial AIF images, + 3 SDs for radial non‐AIF images, and + 2 SDs for qPerf non‐AIF images; (c) suppressing background (e.g., removing high chest‐fat signal above the 95th percentile of baseline) followed by morphological mask refinement with MATLAB's functions such as “*imopen*”. Pixels below the 5th percentile of intensity within the region of interest (ROI) were discarded to exclude muscle signal. Visual inspection was performed for all cases since automatic detection of ROI failures were not implemented. In cases where automatic detection was unsuccessful (1 of 47 radial AIF, 1 of 47 radial non‐AIF, and 1 of 61 qPerf non‐AIF series), ROIs were further corrected manually. Finally, RV and LV signal intensity and [Gd] time curves were normalized to the same maximum to preserve their relative amplitude relationship. For details on converting signal intensity to [Gd], please refer to [26].

**FIGURE 2:**
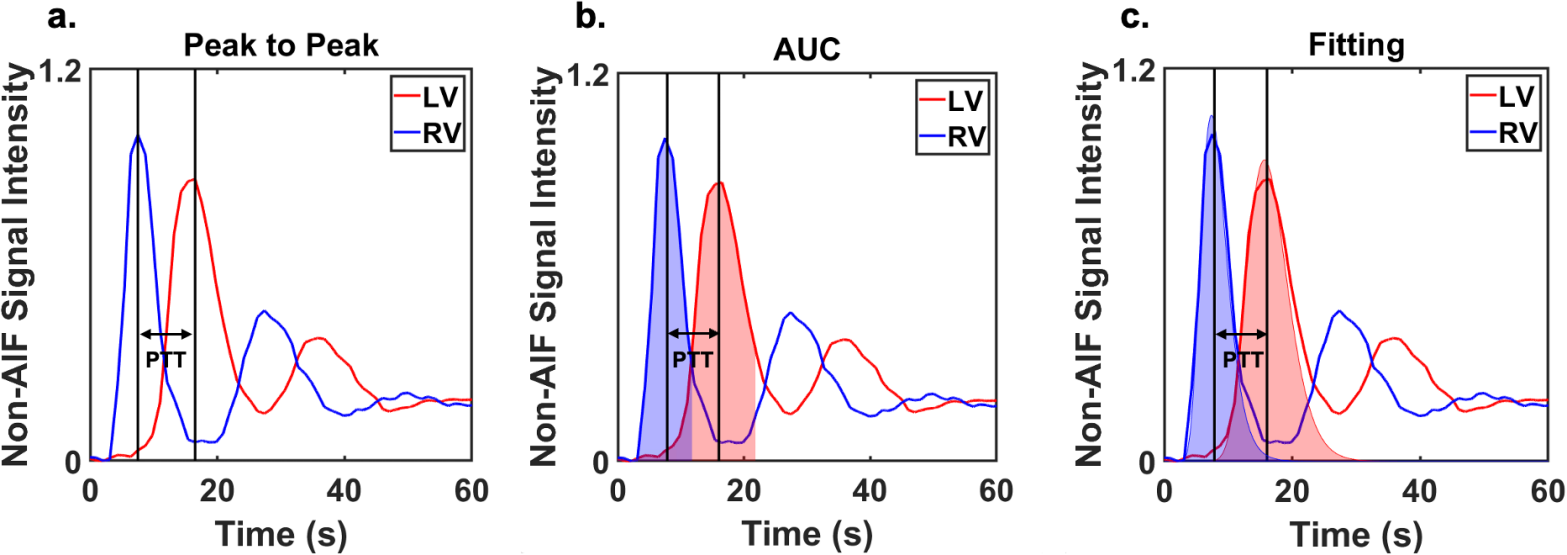
A schematic showing three approaches to calculate PTT: (a) peak‐to‐peak, (b) AUC centroid‐to‐centroid, and (c) lognormal curve fitting centroid‐to‐centroid. AUC, Area under the curve; non‐AIF, Non‐arterial input function images (i.e., myocardial enhancement images); PTT, Pulmonary transit time; LV, Left ventricular; RV, Right ventricular.

For the peak‐to‐peak method (Figure 2a), PTT was defined as the time interval between the peaks of the RV and LV curves. For centroid‐based methods, rather than identifying the first‐pass endpoint, we approximate an endpoint as peak + Δt, where Δt corresponds to the time interval between the signal intensity at 0.2 and the peak. This approach was adopted because the first‐pass endpoint is in some instances difficult to identify (see Figure S1). To enhance the robustness of detecting these points, we interpolated the signal with a time step of 0.01 and applied MATLAB's “smoothdata” function to both the signal and [Gd] time curves. In the AUC method (Figure 2b), the centroid was calculated as the center of mass of the original curve from baseline up to the reference endpoint. For the curve fitting approach (Figure 2c), a three‐parameter lognormal function was fitted to the identical portion of the curve as the AUC method:

$$f\left(x\right)=\frac{a}{x\sigma \sqrt{2\pi }}\;e^{-\frac{{\left(\ln x-\mu \right)}^{2}}{2\sigma^{2}}}$$

with initial parameter set as *a* = maximal of the curve, σ = 2, μ = 2. Based on our observations, varying the initial values of σ and μ does not influence the final PTT measurement once the curve fitting converges successfully (see Figure S2). In practice, setting both parameters to 2 consistently yielded stable and accurate fitting results across all cases. The centroid was then calculated as the center of mass of the fitted curve. The PTT was defined as the time difference between the centroids of the RV and LV curves in both AUC and curve‐fitting methods.

### 2.6 Statistical Analysis

Statistical analyses were conducted by one investigator (L.F.) using the *Statistics and Machine Learning Toolbox* in MATLAB. Bland‐Altman analysis and concordance correlation coefficient (CCC) were used to assess the agreement of PTT measurements derived from different image types. Additionally, Pearson correlation analysis was performed to evaluate the relationship between PTT and mean pulmonary artery pressure (mPAP) in the CTEPH subgroup of cohort 1. A *p* < 0.05 was considered statistically significant for all tests.

## 3 Results

The total PTT calculation times were 0.23 ± 0.06 s for the peak‐to‐peak method, 0.31 ± 0.12 s for the AUC‐based centroid method, and 0.59 ± 0.25 s for the fitting‐based centroid method.

**FIGURE 3:**
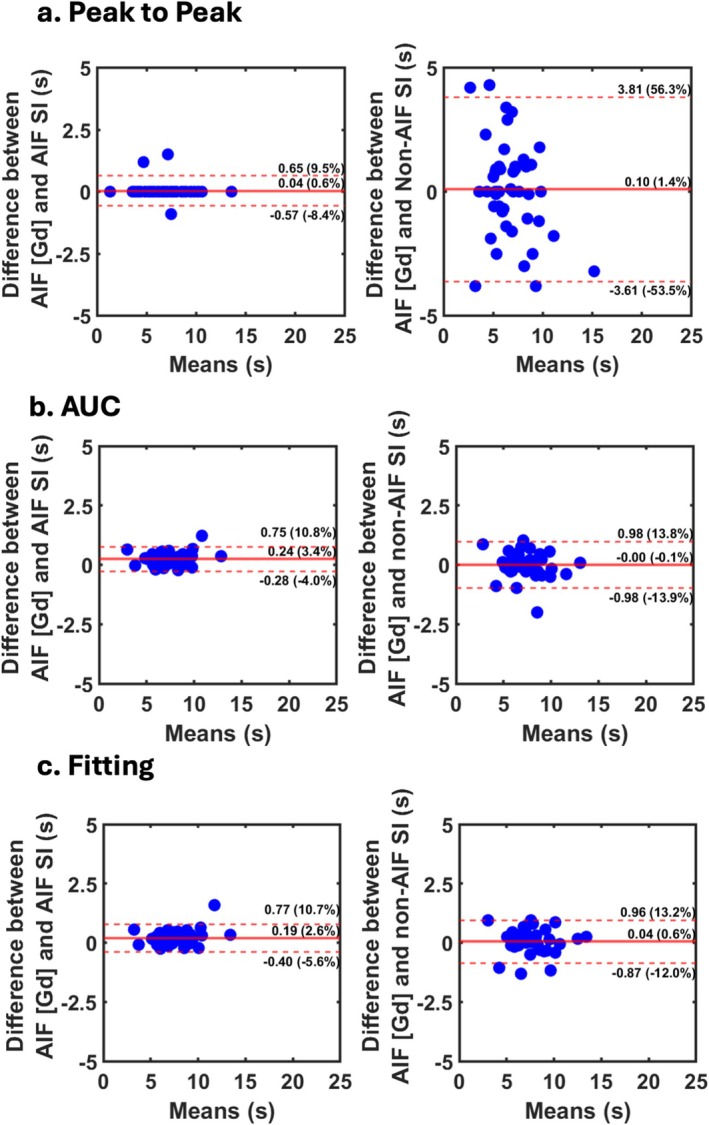
Bland‐Altman plots comparing PTT measurements derived from the radial perfusion pulse sequence: AIF [Gd] (reference), AIF signal intensity, and non‐AIF signal intensity using three methods: (a) peak‐to‐peak, (b) AUC‐based centroid, and (c) curve fitting‐based centroid. AIF, Arterial input function; non‐AIF, Non‐arterial input function images (i.e., myocardial enhancement image); AUC, Area under the curve; PTT, Pulmonary transit time; [Gd], Gadolinium concentration; SI, Signal intensity.

Figure 3 shows the Bland‐Altman plots comparing PTT derived from radial sequence AIF and non‐AIF signal intensities against the AIF [Gd] (reference) using the three methods: peak‐to‐peak, AUC‐based centroid, and curve fitting‐based centroid. For PTT derived from AIF signal intensities, all three methods showed low bias (< 0.25 s [< 3.5% of mean]), narrow ranges of agreement (< 1.25 s [18.0% of mean]), and excellent correlations (CCC > 0.98) compared with the reference PTT derived from AIF [Gd] curves. For PTT derived from non‐AIF signal intensities, the peak‐to‐peak method showed: low bias (0.10 s [1.4% of mean]), a wide range of agreement (7.42 s [109.8% of mean]), and moderate correlation (CCC = 0.70). The corresponding AUC and curve fitting centroid‐to‐centroid methods produced: negligible bias (−0.00 s [−0.1% of mean] and 0.04 s [0.6% of mean] for AUC and fitting, respectively), considerably narrower ranges of agreement (1.96 s [27.7% of mean] and 1.83 s [25.2% of mean] for AUC and fitting, respectively), and excellent correlations (CCC = 0.97 for both AUC and fitting).

**FIGURE 4:**
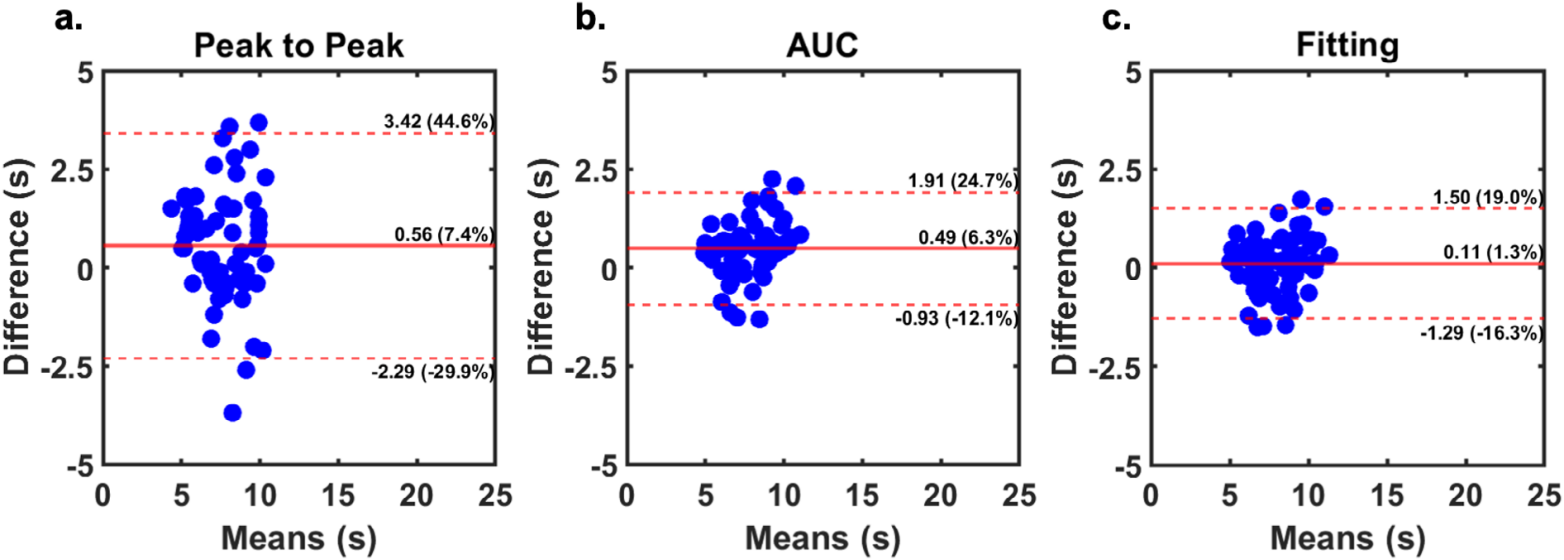
Bland‐Altman plots comparing PTT derived from the prototype qPerf pulse sequence between non‐AIF signal intensities and AIF [Gd] (reference, inline calculation) using three methods: (a) peak‐to‐peak, (b) AUC‐based centroid, and (c) curve fitting‐based centroid. AIF, Arterial input function; non‐AIF, Non‐arterial input function images (i.e., myocardial enhancement image); AUC, Area under the curve; PTT, Pulmonary transit time; [Gd], Gadolinium concentration.

Figure 4 shows the Bland‐Altman plots comparing the qperf non‐AIF signal intensities against the AIF [Gd] (reference) using the three methods: peak‐to‐peak, AUC‐based centroid, and curve fitting‐based centroid. The peak‐to‐peak method produced a moderate bias (0.56 s [7.4%]), a wide range of agreement (5.71 s [74.5% of mean]), and a moderate correlation (CCC = 0.64). The AUC centroid‐to‐centroid method showed smaller bias (0.49 s [6.3% of mean]), considerably narrower ranges of agreement (2.84 s [36.8% of mean]), and a good correlation (CCC = 0.86). The AUC fitting method generated the lowest bias (0.11 s [1.3% of mean]), comparable ranges of agreement (2.79 s [35.3% of mean]), and an excellent correlation (CCC = 0.91).

**FIGURE 5:**
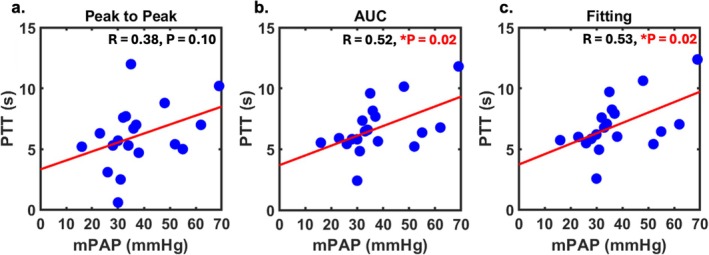
Correlation between PTT and mPAP. PTT values were calculated from non‐AIF signal intensities in the CTEPH subgroup of cohort 1 using three methods: (a) peak‐to‐peak, (b) AUC‐based centroid, and (c) curve fitting‐based centroid. *p < 0.05 was considered statistically significant. Non‐AIF, Non‐arterial input function images (i.e., myocardial enhancement image); AUC, Area under the curve; CTEPH, Chronic thromboembolic pulmonary hypertension; mPAP, Mean pulmonary artery pressure; PTT, Pulmonary transit time.

Figure 5 shows the regression plots comparing the PTT and mPAP derived from non‐AIF signal intensities using the three methods: peak‐to‐peak, AUC‐based centroid, and curve fitting‐based centroid. Significant correlations (*p* < 0.05) were observed for the AUC (*R* = 0.52) and fitting (*R* = 0.53) centroid‐to‐centroid methods.

## 4 Discussion

This study demonstrated that PTT can be measured from non‐AIF first‐pass perfusion images without a significant bias by using either the AUC or curve‐fitting centroid method, even without converting the signal to [Gd]. PTT derived from non‐AIF images using both centroid methods was relatively accurately compared to PTT derived inline from a prototype dual‐imaging pulse sequence.

A previous study suggested that AIF imaging is necessary to avoid signal saturation effects when quantifying PTT [4]. While using an AIF image remains ideal, many clinical imaging facilities lack access to a dual‐imaging perfusion pulse sequence, as not all vendors provide such. In such instances, a solution derived from non‐AIF images may provide a viable alternative. Furthermore, converting signal intensity to [Gd] requires knowledge of specific sequence parameters, which are often unavailable to users who did not develop the sequence themselves. As such, a method that directly uses signal intensity curves from standard non‐AIF perfusion images is more clinically feasible. Our results demonstrated that both centroid‐to‐centroid methods provide relatively accurate PTT measurements (percent difference ranging from −0.1% to 6.3% and CCC > 0.85) and moderate scatter in PTT (25.2%–36.8% range of agreement). Our findings suggest that PTT can be estimated without a significant bias from standard perfusion images without requiring dedicated AIF acquisition. However, the results should be interpreted with caution, as the variability in PTT measurements may be non‐trivial considering the typical range observed between healthy and patient populations. For example, Houard et al. found that PTT was significantly prolonged in heart failure patients with reduced ejection fraction compared to controls (9 ± 6 vs. 7 ± 2 heartbeats) [1]. Similarly, Segeroth et al. reported a median PTT of 6.8 s (interquartile range: 5.9–7.9 s) in subjects with preserved left and right ventricular ejection fractions (> 50%) and 9.3 s in those with reduced left or right ventricular ejection fractions (< 40%) [10].

This study has several limitations that warrant consideration. First, our mixed patient cohort included a limited number of individuals with impaired pulmonary circulation. Future studies should validate our findings in larger populations, with special emphasis on enrolling patients with impaired pulmonary circulation (e.g., pulmonary hypertension, heart failure) are warranted. Second, all data were acquired using two different pulse sequences on one MRI vendor, which may limit generalizability. Future studies should incorporate datasets from different acquisition/clinical protocols and multiple vendors to evaluate clinical broader applicability. Third, the reconstruction time of our radial perfusion images was 840 ± 178 s, which limits its clinical translation. Future work incorporating advanced approaches (e.g., cuFINUFFT [27], Toeplitz [28], and deep learning [29]) is warranted to further accelerate reconstruction and overall workflow efficiency.

In summary, this study demonstrated that PTT can be derived accurately from non‐AIF perfusion images using one of the two tested centroid‐to‐centroid methods, even without converting signal intensity to [Gd].

## Supporting information


**Figure S1:** AIF and non‐AIF signal intensity curves from a representative case showing absence of the second peak, despite acquisition of more than 90 time frames. AIF: arterial input function; non‐AIF: non‐arterial input function images (i.e., myocardial enhancement image).
**Figure S2:** Lognormal curve fitting centroid‐to‐centroid plots from one representative case, illustrating results obtained with different initial values of σ and μ.
**Table S1:** Relevant demographics of two patient cohorts. Bpm, beat per minute; LVEF, left ventricle ejection fraction.
**Table S2:** A summary of pulse sequence parameters. PD, proton density; FOV, field of view; TE, echo time; TR, repetition time; AIF, arterial input function; non‐AIF, non‐arterial input function images (i.e., myocardial enhancement images); b‐SSFP, balanced steady‐state free precession; GRE, gradient‐recalled echo; TS, saturation‐recovery time.

## Data Availability

The MATLAB code for automated PTT calculation will be made available upon reasonable request.
