# Supplementary material for: Pulmonary Transit Time Can Be Accurately Quantified From Non‐Arterial Input Function Series in First‐Pass Cardiac Perfusion MRI
Source: Magn Reson Med. 2025 Nov 23;95(4):2207–13. doi: 10.1002/mrm.70190 (PMC12850576; doi:10.1002/mrm.70190)
Supplement: Supplementary file 1 — Figure S1: AIF and non‐AIF signal intensity curves from a representative case showing absence of the second peak, despite acquisition of more than 90 time frames. AIF: arterial input function; non‐AIF: non‐arterial input function images (i.e., myocardial enhancement image). Figure S2: Lognormal curve fitting centroid‐to‐centroid plots from one representative case, illustrating results obtained with different initial values of σ and μ. Table S1: Relevant demographics of two patient cohorts. Bpm, beat per minute; LVEF, left ventricle ejection fraction. Table S2: A summary of pulse sequence parameters. PD, proton density; FOV, field of view; TE, echo time; TR, repetition time; AIF, arterial input function; non‐AIF, non‐arterial input function images (i.e., myocardial enhancement images); b‐SSFP, balanced steady‐state free precession; GRE, gradient‐recalled echo; TS, saturation‐recovery time. [file MRM-95-2207-s001.docx]

**Supporting Information**

**Supporting Information Table S1:** Relevant demographics of two patient cohorts. Bpm: beat per minute; LVEF: left ventricle ejection fraction.

|  | **qPerf sequence cohort**  **(N = 61)** | **Radial sequence cohort**  **(N = 47)** |
| --- | --- | --- |
| **Age (years)** | 57 ± 17 | 52 ± 17 |
| **Gender (male/female)** | 34/27 | 26/21 |
| **Heart Rate (bpm)** | 73 ± 13 | 75 ± 15 |
| **LVEF (%)** | 54 ± 15 | 57 ± 6 |

**Supporting Information Table S2:** A summary of pulse sequence parameters. PD: proton density; FOV: field of view; TE: echo time; TR: repetition time; AIF: arterial input function; non-AIF: non-arterial input function images (i.e., myocardial enhancement images); b-SSFP: balanced steady-state free precession; GRE: gradient-recalled echo; TS: saturation-recovery time.

|  | **qPerf** | **Radial Perfusion** |
| --- | --- | --- |
| **PD frames** | 3 | 6 |
| **FOV (mm × mm)** | 361×209 to 470x272 | 384×384 |
| **Acquisition matrix size** | 192×111 | 192×192 |
| **k-space lines acquired per image** | 55 | 42 |
| **Acceleration factor** | 2.0 | 4.6 |
| **Spatial resolution**  **(mm × mm)** | 1.88×1.88 to 2.45×2.45 | 2×2 |
| **Slice thickness (mm)** | 8 | 8 |
| **TE (ms)** | 1 | 1.5 |
| **TR (ms)** | 2.48 | 2.8 |
| **Temporal resolution (ms)** | 136.4 | 117.6 |
| **Receiver bandwidth (Hz/pixel)** | 1085 | 745 |
| **AIF flip angle (°)** | 5 | 15 |
| **Non-AIF flip angle (°)** | 20-50 | 15 |
| **AIF sequence type** | GRE | GRE |
| **Non-AIF sequence type** | b-SSFP | GRE |
| **TS for AIF (ms)** | 29 | 10 |
| **TS for non-AIF (ms)** | 105 | 119 |

**
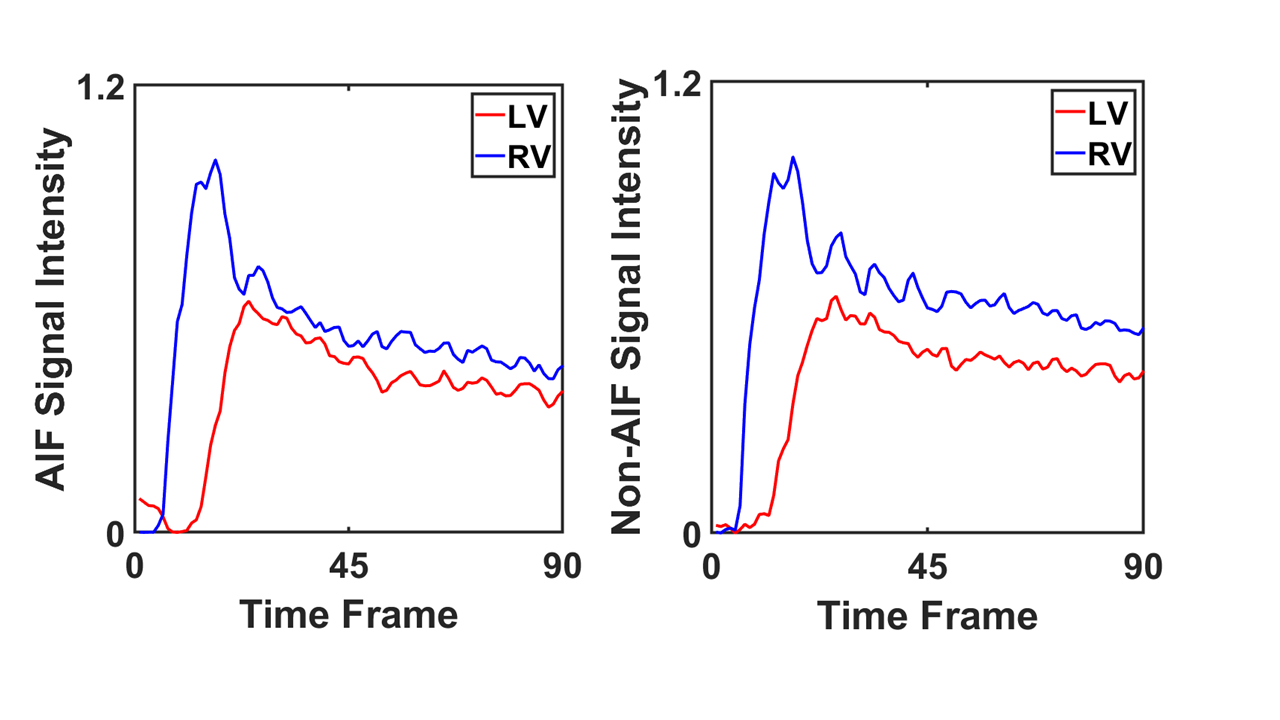
**

**Supporting Information Figure S1:** AIF and non-AIF signal intensity curves from a representative case showing absence of the second peak, despite acquisition of more than 90 time frames. AIF: arterial input function; non-AIF: non-arterial input function images (i.e., myocardial enhancement image).


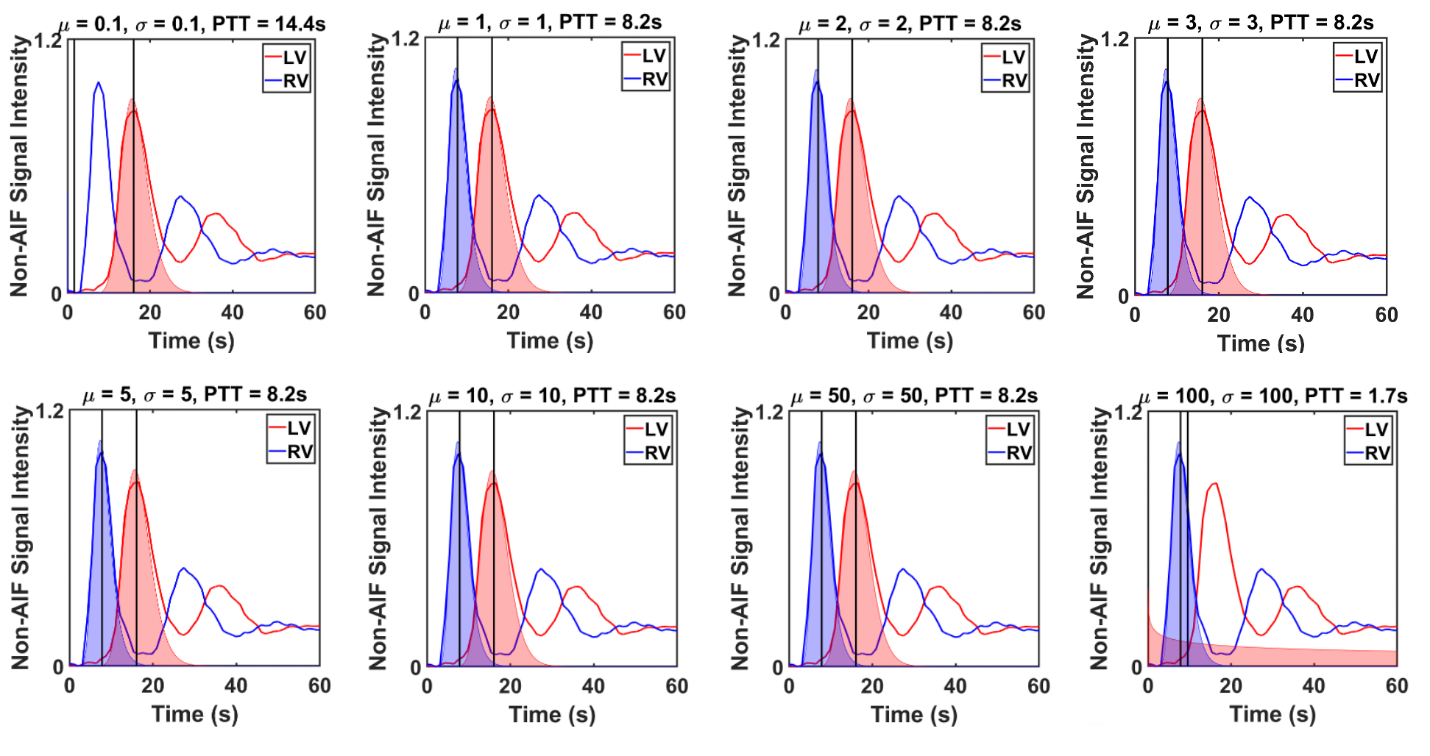


**Supporting Information Figure S2:** Lognormal curve fitting centroid-to-centroid plots from one representative case, illustrating results obtained with different initial values of $\sigma$ and $\mu$.
